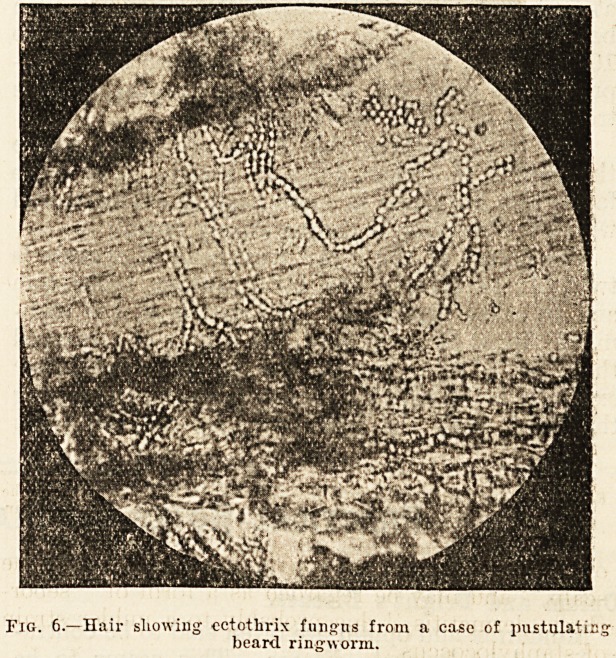# Skin Affections of the Beard Region Due to Micro-Organisms, and Their Treatment

**Published:** 1911-02-04

**Authors:** H. G. Adamson

**Affiliations:** Physician in Charge of the Skin Department at St. Bartholomew's Hospital.


### Hospital Clinics.

### SKIN AFFECTIONS OF THE BEARD REGION DUE TO MICRO-ORGANISMS, AND THEIR TREATMENT.

By H. G. ADAMSON, M.D., Physician in Charge of the Skin Department at St. Bartholomew's Hospital.

The beard region may be the seat of various eruptions as the result of local infection by micro-organisms.

The most important of these are: impetigo contagiosa, sycosis, and ringworm.

None of these affections is of rare occurrence, and they may be seen in all classes of patients. By those unfamiliar with their individual characters they are liable to be mistaken one for another. For their successful treatment it is very important to distinguish them, and the object of this paper is to point out their clinical features and to discuss their diagnosis, with the view to their appropriate treatment.

[F]ig. 1.—Impetigo contagiosa of the beard region, showing the stuck-on crusts (*streptococcic impetigo*).

When a patient presents himself with an eruption on the beard region the first question to ask is as to its duration. If it is of recent origin it is probably an impetigo contagiosa or a ringworm. If of long standing—months or years—generally a sycosis. The characteristic lesions of these eruptions are, respectively, the stuck-on crust of impetigo, the broken hair stump of ringworm, and the follicular pustule of sycosis. It is upon the finding of these lesions that we base our diagnosis and our treatment.

### Impetigo Contagiosa.

Impetigo contagiosa of the beard region is a common affection, the true nature of which is often overlooked, probably because no particular attention is drawn to it in text-books. The importance of an acquaintance with it will be presently stated.

The patient presents, on the beard region, some flattened yellowish crusts (fig. 1), and he sometimes gives a history that they began to appear a few days after he had been shaved by a barber. It will be noted that the crusts are quite easily removed, and that when a crust is picked off with forceps there is left only a superficial, red, moist excoriation. There are no pustules involving hair-follicles. There is no deep-seated inflammation. Careful observation will sometimes reveal, between the crusts, small flaccid, very superficial blisters, which are the primary lesion, the crusts being due to the drying of the serum of such blisters. Often the crusts are not limited to the beard region, but are scattered also over the face. This condition is identical with that seen in children, and called impetigo contagiosa, and it is a streptococcic infection. By a very simple treatment, to be presently described, a case of this sort is easily cured. On the other hand, if it is misunderstood, and not efficiently treated, secondary infection with staphylococcus pyogenes takes place, and a very intractable follicular-pustular eruption results. In fact, a sycosis, the affection which we are about to consider, is produced.

Fig. 2.—Sycosis menti; showing the perifollicular pustules (*staphylococcic impetigo*).

### Sycosis menti.

Sycosis is really a staphylococcic impetigo—the essential lesion of which is a follicular pustule—affecting a particular region. The hair-follicles are the seat of papules and papulo-pustules closely set together over an area of varying extent. There may be a single patch with outlying pustules and papules, or, more often, the whole beard and whisker area, or the moustache region, is involved.

According to the severity of the eruption there is usually more or less deep-seated general infiltration, so that these parts present a uniform redness and swelling studded with pustular points, and here and there crusted (fig. 2). Sometimes the eyebrows, or the margins of the eyelids, and even parts of the scalp, may be involved. On close inspection it will be found that each pustule is centred by a hair, and on pulling the hair with forceps it comes away, together with its swollen root-sheath, allowing a drop of pus to escape. In one type of case the pustulation is much less marked, and there is little more than a collection of follicular papules with scaly tops occupying the affected regions. This milder form

Fig. 3.—Tinea barbæ. The dry scaly form of ringworm, showing the "black-dots" which are formed by broken and curled-up hair-stumps invaded by fungus.

of sycosis is associated with "seborrhœa of the scalp," and may be regarded as a form of "seborrhœic dermatitis," due probably to a milder strain of staphylococcus.

As has already been stated, sycosis is often secondary to a neglected simple impetigo contagiosa, but it may also, in the case of the upper lip, be the result of some chronic nasal inflammation with purulent discharge, a point which should be borne in mind when treating sycosis of the upper lip. Patients afflicted with sycosis are often in a poor state of health from some cause or another, and the worst cases are perhaps seen in hospital patients, who have lived badly through having been out of work. It has already been said that sycosis must be distinguished from impetigo. But it has also to be distinguished from certain types of pustulating beard ringworm, which are described in the next section.

### Ringworm of the Beard: Tinea Barbæ.

This is the disease to which perhaps most often the term "Barber's itch" is applied. It is sometimes called "hyphogenic sycosis," to distinguish it from "coccogenic sycosis"; but the term tinea barbæ is better.

Tinea barbæ is really quite a common affection, but it is, in the milder forms, often overlooked, and in the more severe cases frequently mistaken for sycosis.

There are several clinical types of beard ringworm, which are due to different varieties of ringworm fungus, but it is unnecessary to describe here all of these types or to study very closely the different varieties of fungus which produce them. For practical purposes we may recognise two main groups of beard ringworms. In the first group are dry, scaly forms, with little inflammation in their earlier stages, but sometimes presenting inflammatory nodules later. In the second are very highly inflammatory, pustulating forms, which may often closely simulate staphylococcic sycosis. These two groups of beard ringworms will be best described separately.

The *dry forms of tinea barbæ* are the commoner, but their diagnosis is generally missed by those unfamiliar with their peculiar features. The eruption in its earliest stage consists of reddish, slightly scaly patches and rings situated on the skin of the beard region. At this time the epidermis is alone attacked by the fungus. But soon the hairs become invaded, and then it is that the characteristic features are produced. Some—but usually not all—of the hairs on a scaly patch become broken off quite short. Owing to their want of elasticity—because they are riddled with fungus—the broken off stumps are unable to push their way through the scales over the follicular orifice, and consequently become curled up at the mouth of the follicle. We then see, scattered over a slightly red and slightly scaly area, small black dots, which are the curled up infected hairs, and which can be unravelled with one blade

Fig. 4.—Microscopical appearance of a hair from a case of endothrix beard ringworm (fig. 3), showing fungus inside the hair.

of the forceps (fig. 3). The broken stump, examined in a drop of liquor potassæ under the microscope, is seen to be packed with spores (fig. 4). Sometimes these "black dots," or curled up stumps, are scattered in small groups over the whole beard region.

Occasionally a ringworm of this type will take on an inflammatory condition, so that the beard area is covered with rather deep-seated dusky-red, smooth, flat nodules. The hairs over these nodules are broken and loosened, so that they easily pull out, and often opaque and dull in contrast to the normal hairs. Between the nodules small patches of the black-dot hairs may sometimes still be seen.

The cultures given by hairs from these dry forms of beard ringworm are generally those of the violet endothrix, or of the yellow crateriform endothrix, both varieties which give rise to "large-spored ringworms" on the scalp in children.

*The Pustulating Ringworms of the Beard.*—This type of ringworm is most often met with in those who have to do with animals—cavalry-men, farmers, stable-men, carters. The amount of inflammation varies from a large patch of follicular pustules to a deep-seated brawny carbuncular-looking nodule. Generally the area involved by the inflammatory lesions is much more limited than in sycosis (fig. 5), but in a few cases the whole chin area has been involved by pustules and deep-seated abscesses. On the nodules and in the pustules there are found broken hairs, often swollen and with an opaque ground-glass like appearance, which distinguish them from the loosened hairs of a sycosis. The microscope alone makes the diagnosis sure; but one must be able to recognise the peculiar type of hair-stump with the naked eye, or probably many loosened hairs will be examined

Fig. 5.—One type of pustular ringworm of the beard—a single raised inflammatory pustulating lesion. Other forms simulate sycosis.

before an infected one is found. The stumps found in these cases show the endo-ectothrix type of fungus invasion (fig. 6), and the cultures given by these ringworms are generally exuberantly growing white cultures.

Diagnosis.

Familiarity with the individual characteristics of each of these affections is the surest means to a correct diagnosis. Their main points may be summarised as follows:—

*Impetigo contagiosa* (streptococcic).—History of recent infection. Isolated stuck-on crusts. Absence of follicular pustules or deep-seated nodules. Often crusts of impetigo on other parts of face.

*Sycosis menti* (staphylococcic).—A history of long duration—months or years. Extensive areas of follicular pustulation, either involving the whole of the chin and cheeks, or the upper lip (in connection with a nasal discharge). No ringworm invaded stumps found.

*Tinea Barbæ* (dry form): scaly rings and patches, with broken hair stumps or curled-up hairs. Sometimes smooth indurated nodules with broken stumps.

*Tinea Barbæ* (pustulating form): scaly rings and deeply-seated, inflammatory, circumscribed nodules,

Fig. 6.—Hair showing ectothrix fungus from a case of pustulating beard ringworm.

or more widely inflammatory areas with follicular pustules, in which are found ground-glass like stumps, which show fungus under the microscope.

Tinea barbæ never attacks the upper lip.

Treatment.

The *treatment of impetigo contagiosa* of the beard region is the same as for impetigo elsewhere. The crusts must be removed and a mild antiseptic applied to the exposed raw surfaces. The crusts are best removed by frequent bathing in warm water; a lotion of hydrarg. perchlor. 1 in 8,000 may then be mopped on, and afterwards a little ung. hydrarg. ammoniatæ (gr. x ad ʒj.) applied. In a few days the crusts will cease to form, and in a week the eruption will have disappeared, except for stains, which eventually fade. The importance of treating these cases actively at the first must be again insisted upon, since a secondary staphylococcic follicular infection otherwise takes place, and an obstinate sycosis results.

*Treatment of Sycosis.*—The old method of treating sycosis was by epilation and local antiseptics. The whole of the infected area was gradually cleared of hair by epilation with forceps, and this was a long and painful proceeding. Meanwhile, the area was rubbed daily with a strong antiseptic ointment, an oleate of mercury ointment being a favourite application. By persevering with this treatment for very long periods cures were eventually effected.

The more modern treatments of sycosis are by vaccines and by *x*-rays.

A vaccine of the particular staphylococcus present is made from the cultures obtained from the pustules. A dose of 50,000,000 staphylococci is injected, and the dose repeated at intervals of ten days or a fortnight, *i.e.* so long as the condition continues to improve. In some cases the lesions rapidly dry up after the first few injections. But other cases do not do so well. They may improve at first, but afterwards remain stationary, or even become worse. One may increase the dose to 100 millions, and even to 300 millions; but if improvement does not soon take place the vaccine treatment should be discontinued.

The more unfavourable cases are those where the patient is broken down in health. The more favourable are those in more healthy subjects, and where the eruption is comparatively recent.

The object of the *x*-ray treatment of sycosis is twofold: to remove the inflammatory infiltration and to cause depilation. The details of the technique cannot be given here, but it may be remarked that, as a rule, during the second week after exposure to the rays the sycosis dries up, and during the third week the hair falls, leaving the areas treated bald and free from pustules. But this favourable event does not always occur. In some instances the inflammation is temporarily much increased, and the exacerbation takes several weeks to subside. When it does clear up, however, the skin is left in a much improved state, and sometimes quite free from eruption.

In many cases it is well to combine all these methods, to begin with repeated fomentations with a hot antiseptic lotion or the application of an antiseptic ointment (ung. hydrarg. oleat. 5-10 per cent.), in combination with vaccine treatment. If these fail to cure completely, then the *x*-rays should be used.

### Ringworm of the Beard.

In the markedly inflammatory type of beard ringworm the treatment is simple. All that is needed is the application of an antiseptic ointment, and bathing with an antiseptic lotion. As a lotion, lot. hydrarg. perchlor. 1 in 4,000 may be used. Asan ointment, ung. hydrarg. nitratis ʒj.+resorcin gr. xx.

In the dry scaly forms the cure is less easy. Antiseptic ointments will sometimes cure these cases after several months' daily application, but many cases last longer, even several years. In obstinate cases *x*-ray treatment, as for ringworm of the scalp, should be employed. In any case it is by far the most rapid method, but it is one which is not always available.

## Figures and Tables

**Fig. 1. f1:**
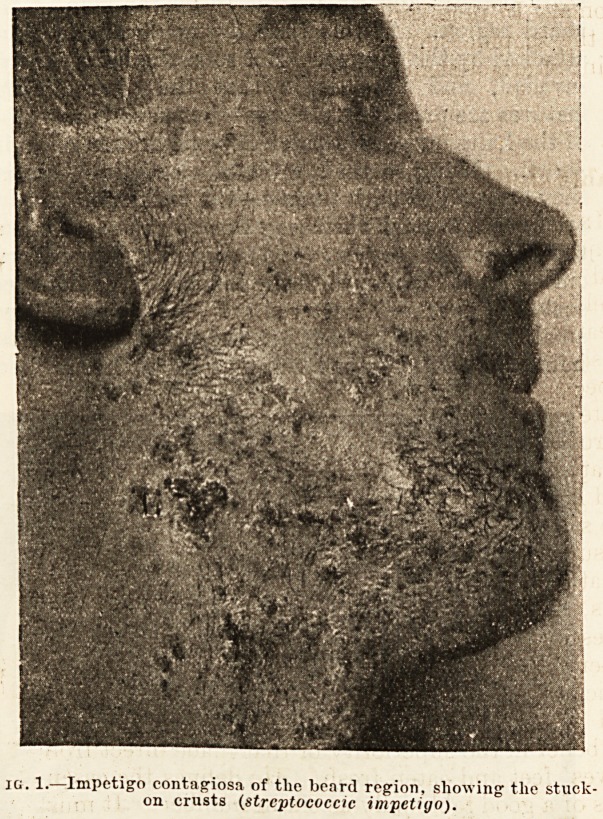


**Fig. 2. f2:**
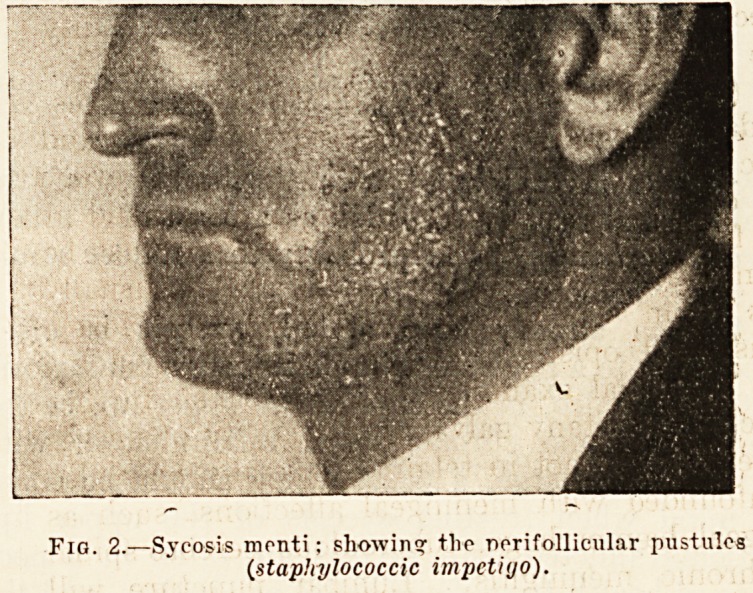


**Fig. 3. f3:**
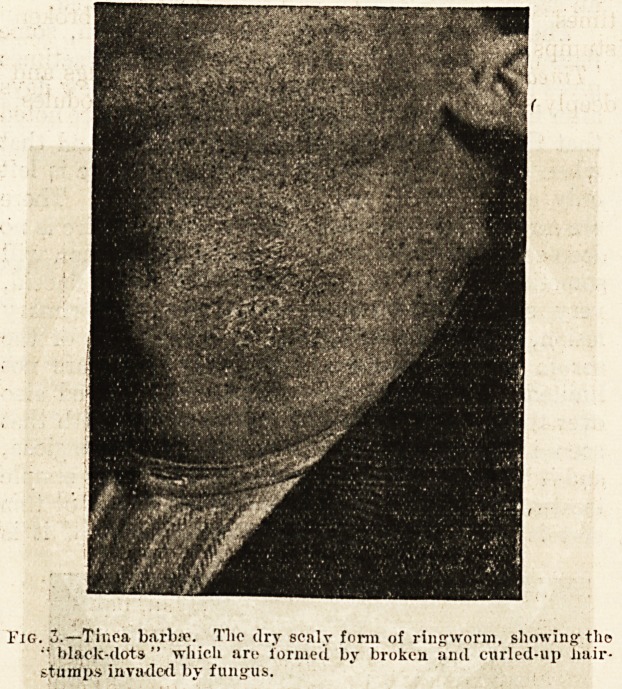


**Fig. 4. f4:**
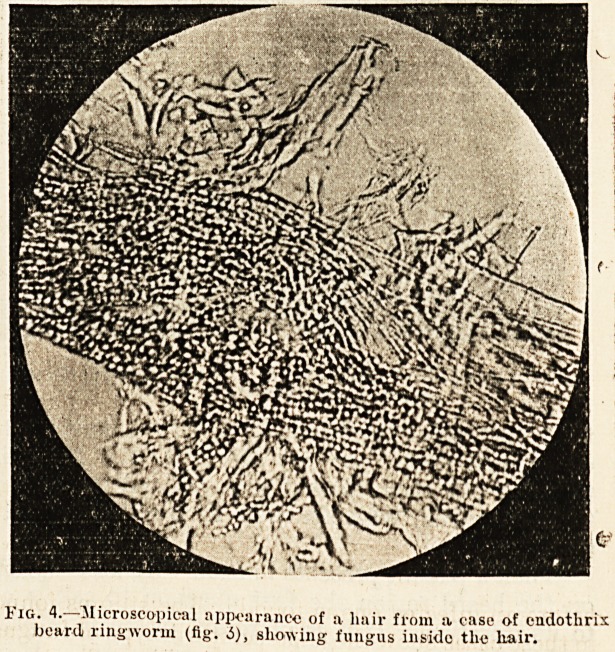


**Fig. 5. f5:**
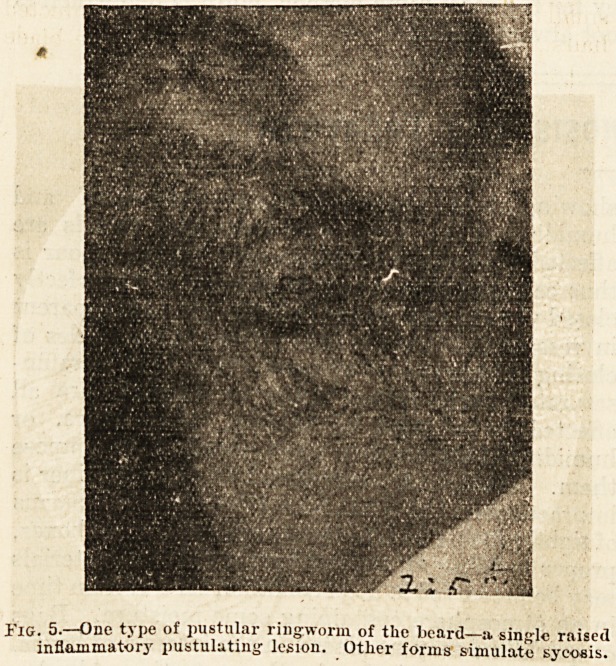


**Fig. 6. f6:**